# Humic Substances in Combination With Plant Growth-Promoting Bacteria as an Alternative for Sustainable Agriculture

**DOI:** 10.3389/fmicb.2021.719653

**Published:** 2021-10-29

**Authors:** Maura Santos Reis de Andrade da Silva, Bianca de Melo Silveira dos Santos, Camilla Santos Reis de Andrade da Silva, Carolina Santos Reis de Andrade da Silva, Luiz Fernando de Sousa Antunes, Roberta Mendes dos Santos, Carlos Henrique Barbosa Santos, Everlon Cid Rigobelo

**Affiliations:** ^1^Department of Agricultural Production Sciences, Universidade Estadual Paulista, Jaboticabal, Brazil; ^2^Department of Soil, Universidade Federal Rural do Rio de Janeiro, Seropédica, Brazil; ^3^National Agrobiology Research Center, Embrapa Agrobiologia, Seropédica, Brazil; ^4^Department of Geochemistry, Universidade Federal Fluminense, Niterói, Brazil

**Keywords:** abiotic stress, biocontrol, endophytic bacteria, fulvic acid, humic acid, nutrient uptake, rhizobia

## Abstract

Plant growth-promoting bacteria (PGPB) and humic substances (HSs) are promising options for reducing the use of pesticides and mineral fertilizers. Although many studies have shown the effects of PGPB and HSs separately, little information is available on plant responses to the combined application of these biostimulants despite the great potential for the simultaneous action of these biological inputs. Thus, the objective of this review is to present an overview of scientific studies that addressed the application of PGPB and HSs to different crops. First, we discuss the effect of these biostimulants on biological nitrogen fixation, the various effects of the inoculation of beneficial bacteria combined with the application of HSs on promoting the growth of nonleguminous plants and how this combination can increase bacterial colonization of plant hosts. We also address the effect of PGPB and HSs on plant responses to abiotic stresses, in addition to discussing the role of HSs in protecting plants against pathogens. There is a lack of studies that address the role of PGPB + HSs in biocontrol. Understanding the factors involved in the promotion of plant growth through the application of PGPB and HSs can assist in the development of efficient biostimulants for agricultural management. This approach has the potential to accelerate the transition from conventional cultivation to sustainable agrosystems.

## Introduction

The use of mineral fertilizers and pesticides ensures high productivity in agricultural systems, and their utilization is justified by the need for increased food production for the growing population. Despite gains in agricultural yields, the use of nonrenewable chemical inputs causes environmental damage, such as contamination of surface water and groundwater and alteration of denitrification processes (Khan et al., 2018). In this sense, the current demands of agriculture are focused on increasing the efficiency of fertilizers and the necessity of more sustainable agriculture as a mode of production. This agroecological model requires not only the use of effective fertilizers in the field but also the use of biostimulants for plant growth, whose role is to improve physiological processes in plants, increase nutrient acquisition, and promote tolerance against abiotic and biotic stresses (Adesemoye and Kloepper, 2009; Olivares et al., 2017; Ekin, 2019).

Plant growth-promoting bacteria (PGPB) constitute an important biological alternative with the capacity to increase yields in several crops. Among the mechanisms involved in promoting plant growth by PGPB is biological nitrogen fixation. Within this process, the most studied plant–bacteria interaction is that of legumes with rhizobia. This symbiosis provides approximately 20 to 22 Tg N per year to agricultural systems. Nonnodulating diazotrophic bacteria also supply N to cultures and associate with a wider range of plants than rhizobia. PGPB are also capable of supplying other nutrients to plants, such as phosphorus (P), through the solubilization of this nutrient through the production of organic acids, H^+^ excreted ions, production of phytases, and release of HCN by PGPB (Backer et al., 2018). Previously, HCN produced by PGPB was associated with the suppression of pathogens; however, Rijavec and Lapanje (2016) proposed that the greatest contribution of HCN by PGPB is sequestration of metals and consequently increased nutrient availability. The production of chelating agents by PGPB can also be associated with a greater supply of Zn and Fe to plants. The production of organic acids and siderophores improves the Fe supply to the plant. Siderophores can also act to inhibit pathogens by depriving these organisms of Fe capture (Ahmed and Holmström, 2014; Shakeel et al., 2015; Backer et al., 2018). In addition, PGPB are capable of producing plant hormones, which in appropriate concentrations, can act on the root and shoot growth of plants and increase root exudation (Maheshwari, 2011; Ruzzi and Aroca, 2015). These microorganisms also improve plant growth under environmental stress, and some studies have shown that PGPB that produce the enzyme 1-aminocyclopropane-1-carboxylase deaminase are able to provide greater tolerance for plants from stress by decreasing ethylene levels, thus preventing a reduction in plant growth. The production of secondary metabolites and volatile organic compounds by PGPB and the induction of gene expression are some of the mechanisms by which PGPB can help plants to more successfully tolerate biotic and abiotic stress conditions (Maheshwari, 2011; Backer et al., 2018). These microorganisms also play a relevant role in biological control. PGPB induce biochemical and plant defense responses, causing induced systemic resistance (ISR; Pereira et al., 2021). In addition to synthesizing compounds that inhibit the growth of pathogens, they can also induce greater production of allelochemicals and change the composition of exudates in plants, contributing to a reduction in pest development (Bashan et al., 2014; Pankievicz et al., 2021).

In turn, humic substances (HSs) are constituent fractions of the organic matter of soil and are highly complex and biologically active (Canellas and Olivares, 2014). These substances include humic acids (HA), fulvic acids (FA), and humin (Schnitzer, 1978). HSs are known to stimulate the root system and plant growth and to mitigate stress damage; their effects extend to soil properties and microbial community structure (Puglisi et al., 2013; Canellas and Olivares, 2014). The action of HSs in the biological activation of plant growth is closely related to their chemical composition (Martinez-Balmori et al., 2014; García et al., 2019). The effect on plant growth of these substances depends on the mode of application of HSs to the plant, content of bioactive molecules, source, dose and molecular weight of the humic fraction, and plant species (Canellas et al., 2010; Nardi et al., 2021). HSs act on the root system, stimulating the quantity and length of lateral roots and root hairs. This process is apparently mediated by HSs in signaling pathways that involve different plant hormones. In addition, HSs play a role in the primary metabolism of plants, acting on the C and N cycles. The effect of HSs on root architecture favors greater soil exploration and, consequently, greater nutrient absorption. The role of HSs in plant nutrition goes beyond the increase in plant root morphology, and these substances can form a complex with cations present in the soil, improving the uptake of nutrients such as P, Zn, and Fe by plants (Olaetxea et al., 2018; Nardi et al., 2021). Furthermore, they are able to affect the expression of nutrient transporters, allowing greater absorption of these elements by plants, in addition, HS may increase the exudation of organic acids from the root, and favoring plant interactions with beneficial microorganisms such as PGPB (Olivares et al., 2017; Nardi et al., 2021). HSs also have the ability to alter secondary plant metabolism, affecting plant genetic expression and inducing the synthesis of compounds that help plants against biotic and abiotic stress (Canellas et al., 2015b; Giovanardi et al., 2016). Salinity and water restriction are the most studied stresses under greenhouse and field conditions, and HSs provide better responses in several agronomic crops against these stresses. The priming effect of HA in corn was generate a decrease in negative impacts of stressors on this plant (Canellas et al., 2020). The action of HSs on the protection of plants against pathogens was documented, and studies have shown that HSs can increase the plant defense system against harmful microorganisms, directly acting in the inhibition of these organisms or inducing the growth of microorganisms with antagonistic action to the pathogen, thus allowing greater protection for the plant (Jindo et al., 2020; Pereira et al., 2021).

Humic substances are relatively recalcitrant to microbial activity and behave as a potential vehicle for these microorganisms. Additionally, HSs have the ability to stimulate the release of organic acids from plant roots, and these compounds represent a source of nutrients for PGPB, which consequently can enhance plant root growth and colonization by these microorganisms, generating several benefits for both plant and soil health (Maji et al., 2017; Olivares et al., 2017; Nardi et al., 2021). Therefore, biological techniques, such as a combination of PGPB and HSs, are promising approaches for improving plant performance and metabolic processes and can reduce financial and environmental costs for agricultural production (Olivares et al., 2017). In this sense, this article aimed to produce an overview of the published scientific studies that have addressed the combination of PGPB and HSs in different agricultural settings. In the first section, we present the role of the simultaneous use of PGPB and HSs in the promotion of plant growth. Then, we demonstrate the actions of these biostimulants in improving the plant response to abiotic stress. Finally, we report the effect of HSs in the protection of plants against pests, mostly against microbial pathogens. Within this context, we also emphasize that few articles have addressed the combined role of PGPB and HSs in combatting this stress.

## Promoting Plant Growth by Humic Substances and Plant Growth-Promoting Bacteria

Plants are highly plastic in development, which affords them sensitivity to respond to the most diverse environmental conditions. The presence of beneficial microorganisms, such as PGPB, and bioactive compounds, such as HSs, has provided the most favorable conditions for various agricultural systems. Some of the significant impacts of PGPB and HSs are improved nutrient acquisition, stimulation of root systems, and greater tolerance to stress (Olivares et al., 2017). Studies with legumes and their symbionts in the presence of HSs have shown promising BNF results. One of the first studies on this theme in a greenhouse showed that the application of Na-humate to soil significantly increased nitrogen uptake, nodulation, and leghemoglobin content of *Sesbania aculeata* inoculated with rhizobia (Gaur and Bhardwaj, 1971). Another study also noted that the application of FA and HA was able to increase the dry weights of plants and nodules in peanuts and soybeans but not the numbers of nodules (Tan and Tantiwiramanond, 1983). Already, Til’ba and Sinegovskaya (2012) observed in the field that application of Na-humate to soybean seeds inoculated with *Bradyrhizobium* in the presence of molybdate, together with foliar application of Na-humate, was able to improve soybean yield in the field; higher values were found for number of nodules and BNF efficiency. The greater efficiency of nodulation in the presence of HSs may be linked to the ability of these substances to regulate quorum sensing (QS) in rhizobia. QS plays an essential role in the growth and development of legume symbiosis (Bogino et al., 2015; Koul et al., 2016).

**FIGURE 1:**
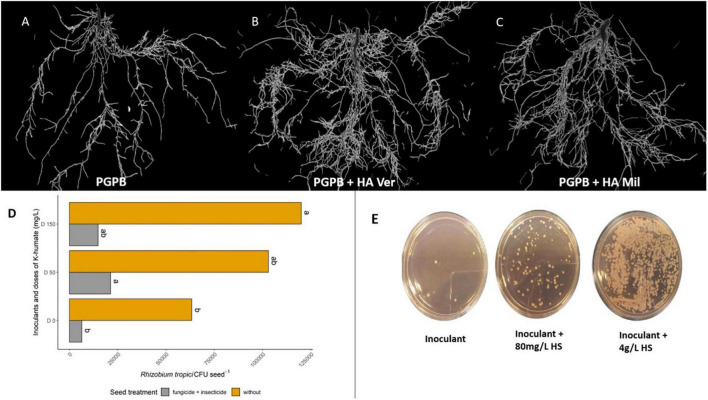
Effects related to the combined use of plant growth-promoting bacteria (PGPB) and humic substances (HSs). **(A–C)** Show the positive impact on the branching and biomass of soybean roots inoculated with *Bradyrhizobium* **(A)** and treated with a combination of humic acid from vermicompost **(B)** and millicompost **(C)** in the soil. **(D)** Shows the increased survival of *Bradyrhizobium* (D0) in soybean seeds in the presence of 50 (D50) and 150 (D150) mgL^–1^ K-humate in both “raw” seeds (yellow bars) and seeds treated with fungicide and insecticide (gray bars). The values are expressed in colony-forming units (CFU) of *Bradyrhizobium* per gram of seed. **(E)** Increased growth of *Rhizobium tropici* (inoculant) with the application of K-humate (unpublished data). Figure created using BioRender (https://biorender.com/).

Humic substances have already been reported to increase microbial growth, affecting the regulation of cellular metabolism (Table 1 and Figure 1) (Kirschner et al., 1999; Tikhonov et al., 2010). One study evaluated the role of water-soluble humic materials in *Bradyrhizobium liaoningense*; under this condition, *B. liaoningense* showed a gene profile similar to that found for the same strain in the presence of flavonoids (Gao et al., 2015). Flavonoids are molecules responsible for activating the expression of genes in rhizobia that are essential for initiating the symbiosis process (Oldroyd et al., 2011). In addition, greater expression of *nod* and *nif* genes and nitrogenase complex formation were observed in free-living bacteria when in contact with this substance. Greenhouse experiments confirmed a greater increase in BNF in plants inoculated with *B. liaoningense* in the presence of water-soluble humic materials. These results elucidated the direct effect of HSs on bacteria and how they may be related to the improvement of symbiosis with the host plant (Gao et al., 2015). FA has also been found to induce the growth of *Sinorhizobium meliloti*. In addition, this combination was shown to provide an increase in active nodules and yield in *Medicago sativa* (Capstaff et al., 2020). These FA-treated plants showed expression in root genes related to various processes, such as defense, oxidoreduction, and C and N metabolism, in addition to specific nodulation genes. These data suggest that HSs act on the plant (Capstaff et al., 2020), inducing early nodulation and regulating the expression of BNF-related genes in microsymbionts (Gao et al., 2015).

**TABLE 1:** Studies examining bacteria associated with humic substances.

**Microorganisms**	**Humic substance**	**Biological effect**	**References**
*Azotobacter chroococcum*	Na-humate and fulvic acid	Na-humate and fulvic acid increase cell growth in *Azotobacter chroococcum*.	Gaur and Bhardwaj, 1971
*Pseudomonas* sp.	Fulvic acid	Fulvic acid increased cell yield and cell yield per μl O_2_ taken up by *Pseudomonas*.	De Haan, 1974
*Klebsiella aerogenes*	Humic acid	Humic acid increased the survival of *Klebsiella aerogenes* exposed to ultraviolet irradiation (UV).	Bitton et al., 1972
*Mycobacterium avium*	Humic acid and fulvic acid soil	Humic acid and fulvic acid stimulated cell growth of *Mycobacterium avium*.	Kirschner et al., 1999
*Bacillus subtilis*	Humic acid	Humic acid increased the number of *B. subtilis* immobilized in alginate beads.	Young et al., 2006
Bacteria in soil and in the digestive tracts of earthworms	Humic acid	Humic acid stimulated bacterial growth.	Tikhonov et al., 2010
*Bradyrhizobium liaoningense*	Water-soluble humic materials (WSHM)	WSHM stimulated cell growth and metabolism, including nodulation-related proteins and BFN.	Gao et al., 2015
*Streptomyces* sp.	Humic acid	Humic acid stimulated both growth and the ability of *Streptomyces* sp. to solubilize rock phosphate.	Farhat et al., 2015
*Sinorhizobium meliloti*	Water-soluble humic materials	WSHM regulate the quorum sensing and increased cell density of *Sinorhizobium meliloti*.	Xu et al., 2018
*Sinorhizobium meliloti*	Commercial fulvic acid	Fulvic acid stimulated cell growth of *Sinorhizobium meliloti*.	Capstaff et al., 2020
*Bradyrhizobium* sp.	K-humate from leonardite	Increased *Bradyrhizobium* spp. survival in soybean seeds.	da Silva et al., 2021b

**FIGURE 2:**
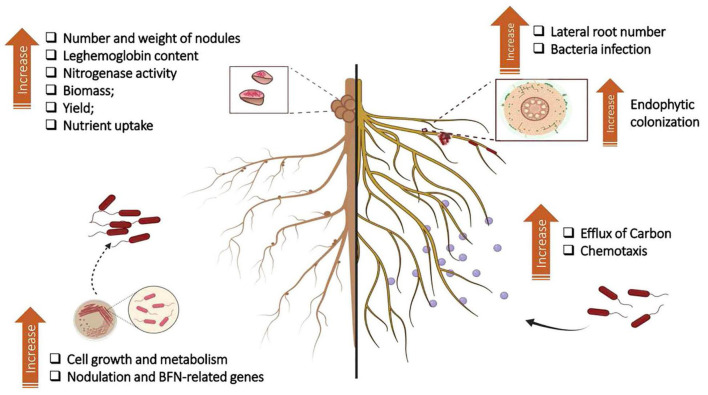
Summary of results found in the literature on the effects of humic substances (HSs) on plant growth-promoting bacteria (PGBP) and interaction with plant root systems. The application of HSs to culture medium affects the growth and bacterial metabolism of rhizobia and induces the expression of genes related to nodulation and nitrogen fixation processes. Experiments in a greenhouse revealed that the application of HSs + rhizobia to legumes provided an increase in the number and weight of nodules, N levels, nitrogenase activity, and leghemoglobin contents. In nonleguminous plants, the combination of HSs + PGPB has been linked to increased endophytic colonization. The increase in root branching due to HSs provides more infection points for bacterial entry and, therefore, for colonization of plant tissue. The increase in carbon efflux through the roots stimulates microbial chemotaxis from the soil to the rhizosphere. Adapted from Olivares et al. (2017). Figure created using BioRender (https://biorender.com/).

The application of K-humate and *Bradyrhizobium* to soybean seeds was found to result in morphological changes in plant roots compared with the inoculated control (Figure 1). In a greenhouse experiment, better values were observed for nodulation and N increase in the shoots of plants inoculated with *Bradyrhizobium* with 50 mg/L K-humate via seeds than in shoots of plants inoculated only with the control (da Silva et al., 2021a). The application of K-humate to chicory generated gains in plant growth and variations in the number of bacterial autotrophic and heterotrophic nitrifiers in the soil. This study suggested that the effect of plant growth and microorganism variation may be related to increased nutrient permeability of the plant membrane. This same work isolated the effect of K on these parameters, stating that the gains obtained came from HSs (Valdrighi et al., 1996). In addition, HSs appear to increase the survival of rhizobia in soybean seeds (Figure 1) (da Silva et al., 2021a), to protect bacteria against irradiation (Bitton et al., 1972), and to increase the viability of the inoculant during storage when applied together with alginate (Young et al., 2006). These characteristics indicate that in addition to improving communication between microorganisms and plants, this combination can protect the inoculant against harmful effects of the environment (Table 1 and Figure 2).

Another study used a phosphorus solubilizing bacteria, *Pseudomonas putida*, together with HA in soybean plants. Despite increasing pH and phosphorus (P) in the soil, the combination was not able to increase crop yield (Winarso et al., 2021). In an experiment using pea as a host plant, it was observed that the application of vermicompost enriched with HA (HARV) was able to provide soil health and plant growth, as well as root nodulation and colonization by arbuscular mycorrhizal fungi (AMF). The authors suggested that AMF and rhizobia act synergistically with HARV on soil and plant improvement (Maji et al., 2017). AMF play an important role in the supply of P to plants (Smith et al., 2011). P is one of the most limiting nutrients for agricultural production due to its low availability in the soil and the high demand of plants for this nutrient in their early stages of growth (Chien et al., 2011). A study using P-solubilizing microorganisms (PSM) and HSs attempted to evaluate the effect of these biostimulants on the P solubility of natural rock phosphate. The results showed an increase in the shoot and root weight of plants compared with the noninoculated treatment. The findings suggested an increase in the efficiency of P use and that the application of PSM and HSs can become an alternative to reduce the use of soluble P fertilizers without harming plant yield (Giro et al., 2016). A recent study also reported that the combined use of HA and *Pseudomonas* spp. and *Bacillus amyloliquefaciens* in maize provided superior effects on P absorption compared with the isolated inoculation of each bacterial strain (Cozzolino et al., 2021). The greatest increase in P uptake was obtained when *B. amyloliquefaciens* was applied in combination with HA and AMF and when *Pseudomonas* spp. was used together with HA. This same work observed significant changes in bacterial and fungal diversity upon inoculation of the strains alone or in combination with HA and AMF. Thus, combinations of biostimulants can promote greater plant growth along with changes in soil microbiota.

The application of FA to *M. sativa* was found to induce the expression of genes involved in cell wall modification (Capstaff et al., 2020). The positive regulation of genes related to cell wall plasticity and root hair differentiation was observed to promote *Arabidopsis thaliana* inoculation with *Azospirillum* (Spaepen et al., 2014). The action of HA on the functionality of the roots and consequent plant growth was found to be related to an initial stage of physical interaction of the molecular complex of HA with pores of the cell wall (Olaetxea et al., 2015). da Silva et al. (2021b) observed the enrichment of bacteria with the ability to produce enzymes that act on the cell wall, such as cellulases and pectinases, in rice roots treated with HA. The increase in these microorganisms may have been related to changes in the cell wall caused by the interaction of HSs with these structures. It is known that the microbiota associated with plants are influenced by external and internal factors that modulate the physiological processes of plants (Taulé et al., 2021), such as HSs. Therefore, it is suggested that changes in plant microbiota caused by HSs can improve plant physiological processes.

For nonleguminous plants, Canellas and Olivares (2014) proposed the concept of biofertilizers based on inoculation with endophytic bacteria in combination with the application of HSs. These substances would provide an increase in bacterial interaction with the plant host, coupled with protection by the inoculant in the hydrophobic domains of HSs. It was observed that after 25 days of contact with the seed, the greatest bacterial survival occurred in the treatment that received HSs (da Silva et al., 2021a). The structural composition of humified organic matter allows adsorption on roots. This characteristic can promote greater contact of the inoculated bacteria on the roots of plants. The increased chances of inoculant settling in the plant tissue allow preselected microorganisms, when applied to the plant, to have an advantage in colonization compared with competitive soil microorganisms (Olivares et al., 2017).

Additionally, the application of HSs can change the root architecture and morphology, inducing the formation of lateral roots and an increase in root hair length and density (Nardi et al., 2017; Olivares et al., 2017; Tavares et al., 2017). Given that the penetration of endophytic PGPB occurs opportunistically, the natural openings arising from the emergence of new roots provided by HSs can favor the entry of these microorganisms into the plant host (Olivares et al., 2017). Canellas and Olivares (2017) observed that HA was able to modulate root border cells and promote greater aggregation and a consequent increase in the population of inoculated bacteria around these structures, thus favoring the infection process. These results may explain previous studies that showed greater colonization of corn roots by *H. seropedicae* in the presence of HSs (Canellas et al., 2013; Canellas and Olivares, 2017).

**FIGURE 3:**
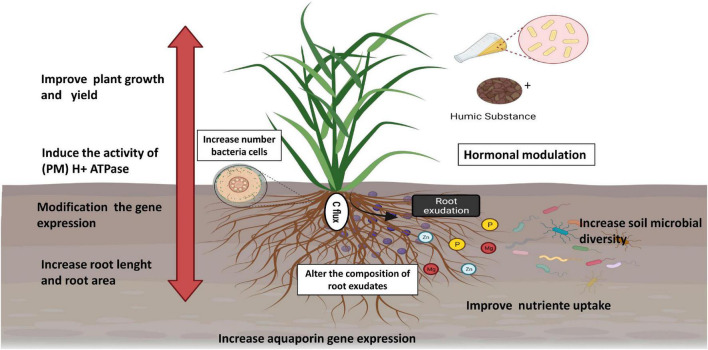
Effect of HSs and PGPB application on plant growth. Figure created using BioRender (https://biorender.com/).

Thus, it is not surprising that PGPB+ HSs promote consistent effects on plant growth, causing increases in the rate of mass increase and higher levels of nutrients (Figure 3). When inoculated with *Bacillus* and HA, tomato plants showed higher levels of Fe and K, in addition to greater growth of shoots and roots (Galambos et al., 2020). The application of HA and *Burkholderia* increased shoots and roots and N, P, K, Ca, and Mg levels in pineapple (Baldotto et al., 2010). In soils with low fertility, treatment with HA and *H. seropedicae* resulted in higher corn production than in the control (Canellas et al., 2015a). In sugarcane, foliar application with *Herbaspirillum seropedicae*, *H. rubrisubalbicans*, and *Gluconacetobacter diazotrophicus* combined with K-humate promoted 37% stem yield over untreated plants (da Silva et al., 2017). Compared with the control, inoculation with *Bacillus megaterium* and *Bacillus subtilis* in combination with the application of 400 kg ha^–1^ HA in a potato plantation yielded an increase in total tuber production of 140%, while the application of 100% NPK fertilizer promoted an increase of only 111% (Ekin, 2019). It is known that soil management practices affect the organic production of vegetables (da Silva C. S. R. A. et al., 2021), and the combined use of HSs and beneficial microorganisms can assist in the transition from conventional cultivation to agroecological practices.

The elevated plant nutrient concentrations are related not only to chelation by PGPB or HSs but also to improvement in the capacity of roots to capture nutrients from soil solution (Zanin et al., 2019; Jindo et al., 2020). These biostimulants can also improve plant nutrition by changing root morphology (Marques Júnior et al., 2008), and this effect is generally attributed to the action of auxins. PGPB can synthesize these hormones, as already demonstrated by HPLC testing (Nutaratat et al., 2017), while HSs can act to mimic these compounds. Studies have also pointed to the presence of auxins in the molecular structure of HSs (Muscolo et al., 1998). In addition, genes related to the transport of macro- and micronutrients were found to be regulated in tomatoes after the application of PGPB and HA and the regulation of genes encoding ATPase (Galambos et al., 2020). Plasma membrane (PM) H+-ATPase is involved in fundamental mechanisms of plant development. For example, (a) the primary ion transport system is essential for nutrient absorption and (b) the growth of a plant cell through acidification of the apoplast is essential for increasing the plasticity of the wall (Hager et al., 1991; Barbez et al., 2017). Knowing that HSs induce the activity of (PM) H+ ATPase in plants, Olivares et al. (2017) hypothesized that the decrease in pH caused by the extrusion of protons by H+-ATPase induced by HSs could facilitate the process of endophytic colonization since the activity of the cell wall-hydrolyzing enzyme used by PGPB for entry and dissemination in plant tissue seems to be increased at low pH.

Changes in plant metabolism after the application of biostimulants have been previously reported. Inoculation with PGPB and HA changed the metabolite fingerprints in maize and sugarcane seedlings. Changes in primary and secondary metabolism were partially related to biostimulation effects on plants (Canellas et al., 2019). The activity of enzymes associated with N assimilation was promoted by applying HA and *H. seropedicae* in maize (Canellas et al., 2013). Similarly, inoculation with *Enterobacter* sp. 32 A, combined with HA application in tomatoes, was able to induce genes related to the assimilation of N (Galambos et al., 2020). The application of beneficial bacteria and humates increased productivity in tomatoes and stimulated secondary metabolism and plant defense (Olivares et al., 2015). The expression of genes related to plant hormones, such as jasmonic acid, auxins, gibberellins, and cytokinins, was observed in plants in the presence of HA and PGPB (Galambos et al., 2020). Hormonal modulation in plants is one of the mechanisms related to the promotion of plant growth. Plant oxidative metabolism also changes in the presence of HA. These compounds were able to induce the production of reactive oxygen species (ROS) and enzymes involved in ROS metabolism in rice roots. Furthermore, the application of HA was correlated with the increased root growth of these plants (García et al., 2016). Studies have proposed that adequate concentrations of ROS can act on root growth signaling (Šamaj et al., 2004). Similarly, the application of pyocyanin, a virulence factor of *Pseudomonas aeruginosa*, at adequate doses, was able to modulate the concentrations of hydrogen peroxide and superoxide in the roots, which in turn seemed to have played a relevant role in altering the root architecture and phytostimulation (Ortiz-Castro et al., 2013).

Physiological changes caused by PGPB and HSs may justify changes in the root exudation profile. The application of *H. seropedicae* and HA altered the composition of root exudates in maize, promoting an increase in the amount and variety of nitrogen compounds (da Silva Lima et al., 2014). Puglisi et al. (2008, 2009) showed that HSs affected the deposition of C by the roots of maize, also resulting in a change in the microbial community of the rhizosphere. The application of HA and a microbial consortium (bacteria and fungi) in blueberries (*Vaccinium corymbosum* L.) significantly altered the bacterial community of the rhizosphere, possibly due to changes in the pattern of root exudates. Coupled with this, an increase of 50% in dry matter of the shoot and 43% of the root was observed (Schoebitz et al., 2016).

Rhizodeposition affects the activity and composition of microbial communities associated with plants. These microorganisms play a fundamental role in plant–soil feedback. The intensification of root exudation of organic acids in maize was reported due to the application of HSs. These organic acids, in addition to assisting in the availability of poorly soluble nutrients, constitute one of the primary sources of C for soil microorganisms, and changes in the pattern of root exudation by HA can lead to an increase in the chemotaxis of these microorganisms to the plant. Furthermore, carboxylates can change the arrangement of HSs, releasing bioactive molecules that generate root modifications and providing entry routes for the colonization of these microorganisms in plants (Olivares et al., 2017; Nardi et al., 2021). Therefore, in addition to microorganisms applied together with HSs having an advantage over soil-dwelling microorganisms, changes in the exudate pattern may contribute to increased soil diversity, recruiting microorganisms that act to improve plant nutrition, protect against stress, and enhance the cycling of nutrients (Figure 3).

## Humic Substances and Plant Growth-Promoting Bacteria Improve Plant Growth Under Abiotic Stress Conditions

Agricultural crops are exposed to multiple stresses throughout their life cycle, which can trigger a decline in productivity and affect food production (Savvides et al., 2016). Integrated models of climate change and agricultural production have projected a decrease in the productivity of crops such as rice, wheat, and corn. Abiotic stress in plants refers to unfavorable weather and/or soil conditions that affect cellular homeostasis and, in more severe cases, impair plant growth and aptitude. These stresses include ion toxicity or deficiency, water surplus or scarcity, exposure to ozone, and extreme temperatures (Mickelbart et al., 2015). Oxidative stress is present in almost all environmental stresses that accompany plants, whether biotic or abiotic, and occurs due to the accumulation of ROS. To prevent ROS damage, enzymatic and nonenzymatic antioxidant compounds are produced to protect the cell (Savvides et al., 2016). Salinity stress, drought, and low temperatures generate osmotic stress, which can lead to loss of cell turgor and decline in plant growth. In part, plants sustain their osmotic homeostasis through the accumulation of osmoregulatory compounds, such as proline, soluble carbohydrates, soluble proteins, and other amino acids, attempting to maintain cellular and plant turgor and thus allowing photosynthesis and plant growth to function. In addition to osmotic stress, salinity causes ionic stress that triggers the accumulation of Na^+^. One of the mechanisms for greater tolerance to this stress is related to the regulation of homeostasis between Na^+^ and K^+^ and the increase in the activity of antioxidant enzymes (Aghaei et al., 2009; Mickelbart et al., 2015; Savvides et al., 2016).

**FIGURE 4:**
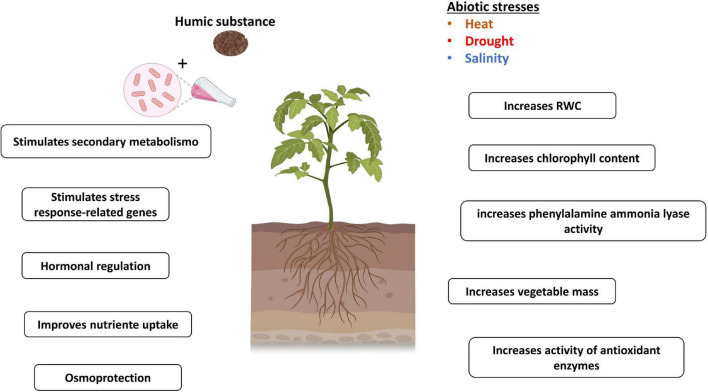
Mechanisms related to plant tolerance to abiotic stress mediated by HSs and PGPB. Figure created using BioRender (https://biorender.com/).

The tolerance of plants to abiotic stress is obtained through different procedures, such as plant breeding, genetic engineering, and fertilizers. These processes are time-consuming and costly and, in some cases, cause damage to the environment (Eneji et al., 2008; Khan et al., 2017; Hasanuzzaman et al., 2018; Lamaoui et al., 2018; Wani et al., 2020). An environmentally suitable approach for improving agricultural production that is capable of mitigating the adverse effects of environmental stresses is the utilization of PGPB and biostimulants, such as HSs (Canellas and Olivares, 2014; Aguiar et al., 2016; Choudhary et al., 2016; García et al., 2016; Olivares et al., 2017; Khan et al., 2020). Biotechnologies concerning this issue are widespread and widely accepted in several locations (Rose et al., 2014; Santos et al., 2019; Khan et al., 2020). The use of PGPB and HSs together or separately has been reported to increase plant growth under normal and stressful conditions (Figure 4) (Canellas and Olivares, 2014, 2017). Previous reports have indicated that changes in HS-induced root morphology can improve PGPB colonization, favoring their fixation and survival on the root surface (Canellas et al., 2013; Canellas and Olivares, 2017). The increases in colonization by PGPB and their multiplication in plants mediated by HSs may be linked to improvement in plant establishment. However, one should not disregard the direct effect of HSs on both plants and inoculated bacteria. A recent study reported that the combined use of *Paraburkholderia phytofirmans*, *Pantoea agglomerans*, *Enterobacter* sp., and HA promoted tomato growth, even with colonization by bacterial strains similar to those on control plants (Galambos et al., 2020).

Recently, the co-application of HA and *Bacillus cereus* SA1 was found to induce morphological and physiological changes in tomatoes in a greenhouse under thermal stress. The plants treated with these two biostimulants under both normal conditions and thermal stress, when compared with the control (without SA1 and HA), showed greater shoot and root lengths; higher concentrations of P, K, and Fe; more noticeable dry and fresh biomass; increased chlorophyll fluorescence; and alterations in the content of salicylic acid (SA; Khan et al., 2020).

Field tests with safflower (*Carthamus tinctorius* L.) showed that the application of different species of *Bacillus* (*B. megaterium* M3 and *B. subtilis* OSU142) in combination with HA under irrigated and nonirrigated conditions displayed an additive effect on the growth and production of this crop (Ekin, 2020). Under rainfed conditions, the combined use of PGPB and HA helped acclimatize safflower plants to a lack of water, improving characteristics related to yield and morphology, such as plant height, number, diameter of chapters, stem diameter, weight of 1,000 seeds, number of seeds and oil (Ekin, 2020). The authors proposed that the gains in rainfed yields may have been related to the greater tolerance of plants to water deficits, with an increase in the content of water and minerals, which could be attributed to the increase in the root system due to the effects of PGPB and HA (Canellas et al., 2002; Zandonadi et al., 2007; Verbon and Liberman, 2016; Ambreetha et al., 2018; Tavares et al., 2020). Similarly, the effect of applying PGPB and HSs on maize was accentuated in a year marked by severe drought stress. It was also noticed that the parcels of the maize that received the biostimulants showed higher amounts of roots left in the soil after harvest, which can contribute to the maintenance of soil organic matter (Canellas et al., 2015a; Olivares et al., 2017).

One of the main impacts of PGPB and HSs on plant growth is the improvement in the acquisition of nutrients and stimulation of the root system. Frequently, these effects are related to auxin or the “auxin-like effect” (Canellas et al., 2002; Zandonadi et al., 2007; Spaepen et al., 2014; Rodrigues et al., 2016). It has been proposed that the role of auxin in plant growth is related to the induction of (PM) H+-ATPase activity, which generates apoplastic acidification and thus provides loosening of the cell wall, allowing cell elongation (Hager et al., 1991; Frías et al., 1996). The activation of (PM) H+-ATPases also improves the absorption of nutrients, boosting the transport of ions across the cell membrane (Sze, 1985; Morsomme and Boutry, 2000). Canellas et al. (2013) reported that the application of *H. seropedicae* and HSs in maize activated metabolism, including the improvement of (PM) H+-ATPase activity, changes in sugar and N metabolism, photosynthesis, induction of lateral root growth, and the most significant colonization by *H. seropedicae*. The application of HSs to wheat plants reduced the toxic effect of the herbicide, stimulated the availability of nutrients in the soil, and promoted gains in productivity. The authors suggested that the positive effect of HSs on P mobilization may have been related to the activation of the rhizosphere microbiota through root exudates (Bezuglova et al., 2019).

Damage to the absorption of nutrients, especially N, and interference with photosynthesis are some of the means by which salt stress interrupts physiological processes (del Amor and Cuadra-Crespo, 2011). PGPB and HSs in different agricultural systems have already stimulated these processes. The application of *Pseudomonas stutzeri* and/or HA reduced salinity damage in peppers under controlled experimental conditions, and the field effect was insignificant and did not reflect productivity gains (Bacilio et al., 2017). This demonstrates the need for field tests to validate the potential of these biostimulants under real planting conditions.

In contrast, the application of PGPB and HA was promising during high-temperature stress in tomatoes; in addition to stimulating plant growth, there was a positive effect on the transcription factor response to thermal stress (*SlHsfA1a*) and the high-affinity potassium (K) transporter (*SlHKT1*; Khan et al., 2020). K assists in regulating ionic balance and in the maintenance of ion homeostasis under salt stress (Hasanuzzaman et al., 2018). PGPB and HA stimulated higher proline concentrations during stress, and the accumulation of amino acids is considered an adaptive mechanism under abiotic stress (Ashraf and Foolad, 2007). Antioxidant enzymes were also stimulated by applying PGPB and HA together with lower values of lipid peroxidation (Khan et al., 2020). These enzymes act to eliminate ROS, compounds that in excess cause damage to plants (Arora et al., 2002). The concentrations of abscisic acid were found to decrease in shoots, while SA was observed to increase in plants (Khan et al., 2020). Studies have also pointed out that the accumulation of SA provides an increase in tolerance to thermal stress in different agricultural systems. Wang et al. (2016) observed that a better response to abiotic stress in apple trees was related to SA stimulus and ROS production. Notably, the protective action of HA in rice plants subjected to osmotic stress was associated with a significant accumulation of ROS in the roots and increased activity of antioxidant enzymes, superoxide dismutase, and peroxidase; in addition, the authors suggested that ROS modulated by HA can regulate cell elongation and differentiation in the root regions, which would justify the greater root stimulation in plants that received HA (García et al., 2016). The evaluation of the recovery of sugarcane plants from water deficit when treated with PGPB and HA revealed that HA helped the recovery of plants from drought through greater induction of antioxidant enzyme activity; this treatment also provided an increase in the rate of liquid photosynthesis because of greater transpiration and stomatal conductance (Aguiar et al., 2016). This behavior was also observed previously in maize under conditions of adequate watering (Canellas et al., 2013). Russell et al. (2006) attributed stomatal opening to the activity of (PM) H+-ATPase induced by the auxin-like action of HSs. While PGPB inoculation induced greater water preservation in plant tissue by maintaining leaf water potential and relative water content (RWC), these effects may be associated with efficient stoma closure. This mechanism is similar to “delayed stress onset,” which is a term used to designate the greatest water preservation in plant tissue (Aguiar et al., 2016). The inoculation of *H. seropedicae* and HA in maize promoted, in relation to the control treatment, an increase expression of the aquaporin gene, *ZmPIP1* (de Azevedo et al., 2019). de Azevedo et al. (2019) suggested that inoculation with *H. seropedicae* and HA can help plants deal with impacts from N reduction and water deficit. Sugarcane plants treated with HA combined with PGPB exhibited the greatest water potential after rehydration and high RWC (Aguiar et al., 2016). Common bean plants cv. Grafite that received applications of *Rhizobium tropici*, *H. seropedicae*, and HSs were subjected to drought; these plants exhibited greater water preservation in plant tissues than control plants due to the greater RWC (da Piedade Melo et al., 2017). In addition, treatments with PGPB + HSs affected secondary plant metabolism, with greater induction of phenylalanine ammonia-lyase activity. The effect of HSs on secondary metabolism was previously reported by Schiavon et al. (2010) in maize plants without stress.

The application of microbial suspensions and HSs can promote the adaptation of plants to stressed environments (Olivares et al., 2017). The use of these compounds in promoting plant growth generates physiological, transcriptional, and metabolic changes that can prepare plants for improved defense before any occurrence of stress (Canellas et al., 2020; Khan et al., 2020). A recent study demonstrated the priming effect of HA in maize and the consequent alleviation of the negative impacts of various abiotic stresses in this plant (Canellas et al., 2020).

## Humic Substances and Plant Growth-Promoting Bacteria in Biocontrol

The application of HSs and PGPB is beneficial for plant health, environmentally friendly for the environment, promotes plant growth, and stimulates defense mechanisms against abiotic and biotic stresses (Giovanardi et al., 2016; Olivares et al., 2017). Some studies have suggested that the application of HA to plant roots generates mild and transient stress, which in turn could promote benefits to plant growth and subsequent resistance to other abiotic stresses (Wilkinson and Davies, 2002). The action of HA on plant growth could be associated with the evolution of stress resulting from the interaction of this compound with root cell walls. The intensity of the stress would depend on the applied HA concentration and its effect on the plant. Lower concentrations would cause mild stresses and stimulate plant growth, while high concentrations would cause intense stress and hinder plant growth. This hypothesis is consistent with that reported by Berbara and García (2014). The authors observed an increase in ROS production in rice (*Oryza sativa*) roots caused by HA application. This action, in turn, was associated with the protection of plants against abiotic stresses (Berbara and García, 2014). However, ROS production is also an important plant defense signal for all types of responses to pathogen attack (González-Bosch, 2018). Few studies have addressed the priming effect of HSs on the induction of plant resistance to biotic and abiotic stresses (Canellas et al., 2020; da Silva et al., 2021b). da Silva et al. (2021b), when investigating the effect of HA on the bacterial community in rice, observed that the microbial groups that were enriched in the presence of HA seemed to be related to plant defense against pathogens; among the microorganisms that increased were members of *Chitinophaga* and *Pseudomonas*, which have recognized roles in biological control (Niu et al., 2020). da Silva et al. (2021b) suggested that mechanisms triggered by HA activate an “alarm” state in a plant, generating a set of adaptive metabolic changes that would allow the plant to respond more effectively to subsequent stresses. Such changes would be responsible for modulating the microbial community in the plant with potential for biological control, recruiting microorganisms that would act in plant defense. Inoculation of tomato with PGPB and HA regulates genes related to plant protection, oxidative stress, and chitin metabolism even under nonstressful conditions (Galambos et al., 2020). These metabolic changes may prepare plants to be more successful at tolerating future biotic and abiotic stress conditions. In another study with tomato, it was observed that the application of PGPB and HSs stimulated secondary and plant defense metabolism (Olivares et al., 2015). Plant secondary products, such as phenolic compounds, are relevant factors for plant resistance and have adverse effects on insect growth and feeding behavior (Cipollini et al., 2008). The application of PGPB or the combination of PGPB and HA was able to induce resistance in canola (*Brassica napus* L.) to the cabbage aphid *Brevicoryne brassicae* L., which is a pest that considerably reduces canola yields both by sucking sap from the phloem and by transmitting phytopathogenic viruses. Higher values of total phenols, flavonoids, and glucosinolates were obtained in canola plants that received PGPB and HA + PGPB. *B. brassicae* had lower longevity and fecundity and a shorter reproductive period in plants treated with these biostimulants than in control plants (Sattari Nasab et al., 2019). Giovanardi et al. (2016) confirmed through transcriptomics that the application of glucohumates (FA + HA) acts as an inducer of resistance to *Xanthomonas arboricola* pv. *pruni* in peach. Among the genes induced in the presence of HSs are those involved in the plant’s direct response to pathogens, with a role in the detection of pathogen-associated molecular patterns, in addition to genes involved in the regulation of redox balance, responses to biotic and abiotic stress, and protection against oxidative stress. This study also found a bacteriostatic/bactericidal effect of glucohumate against *Xanthomonas arboricola* pv. *pruni*. The application of FA to table grapes demonstrated the ability to reduce *Botrytis cinerea* disease through phenylpropanoid metabolism; FA did not show suppressive activity on the growth of mycelium or germination of *B. cinerea conidia* (Xu et al., 2019).

Loffredo and Senesi (2009) reported that other HAs showed suppressive activity against two phytopathogens, *Pythium ultimum* and *Fusarium oxysporum* f. sp. *callistephi*, mainly at the highest concentration. However, they did not observe a significant correlation between the intensity of the inhibitory action on the two phytopathogens and the chemical and functional properties of HA. There was only a negative correlation between *P. ultimum* inhibition and oxygen concentration. This suggests that more factors are involved in these phenomena, such as characteristics inherent to the fungus and environmental conditions. HA application in soybean seeds acted to induce resistance of these plants so that the treatments with HA managed to protect the plants from damping-off and wilt diseases compared with the control condition. In addition, HSs indirectly act to combat pests by improving the establishment and increasing the concentration of microorganisms with biological control action, such as *Trichoderma* (Jindo et al., 2020). The application of HSs in basil was able to influence the antioxidant properties of essential oil, as well as its antibiotic activity against bacteria such as *Klebsiella pneumoniae*. This effect can be attributed to the ability of HSs to affect secondary plant metabolism, promoting a higher yield of phenolic compounds and, consequently, a greater antimicrobial activity of essential oils, which is afeature of great interest for biological control (Verrillo et al., 2021). The application of HA also suppressed the injury caused by grape nematodes, decreasing nematode fertility and fecundity (Kesba and El-Beltagi, 2012). In this same work, the increased productivity of grapes by HA was attributed to an increase in antioxidant compounds and enzymes (Kesba and El-Beltagi, 2012).

The individual use of these biostimulants in biocontrol has already been established in the literature; however, there have been few reports on the mechanisms involved in the interaction of these biostimulants on the protection of plants against pathogens (Glick, 2015; Jindo et al., 2020; Pereira et al., 2021). We hypothesize that HSs and PGPB can use different approaches to contribute to the plant’s defense mechanisms, such as (1) combined application of these biostimulants is capable of altering secondary plant metabolism and pathways related to stress responses, providing the plant with greater tolerance to biotic stress. (2) HSs are able to stimulate bacterial growth and provide physical protection to these microorganisms and this effect may favor the growth of microorganisms related to biological control. (3) The application of PGPB has already been reported to cause changes in the root exudation profile, inducing the production of compounds that are less attractive and even repellent to the pathogen, and in fact, changes in the exudate pattern by HSs have also been documented. Therefore, HSs and PGPB can act directly in the inhibition of pathogens through changes in the composition of root exudates. (4) Application of HSs and PGPB has a direct effect on antagonism to pathogens. (5) Application of HSs can cause increased adherence of bacteria preselected with the ability to suppress pathogens in plant roots, making the potential for inoculation greater and more efficient. (6). More than one of these processes may be occurring at the same time.

## Conclusion

The studies in this review show the potential for the combined use of HSs and PGPB as agricultural inputs in different crops. The literature also reveals similarities in the responses of plants and these different biostimulants, such as root increase, absorption of nutrients, and minor damage to stress. Research using transcriptomic and proteomic techniques is promising to clarify how HSs and PGPB act in plant physiology. These studies can also identify biostimulation markers in plants; such features can assist in genetic manipulation in plant-breeding programs seeking to increase the response of plants to biostimulants. An area that is still little explored is the use of HSs and PGPB in pest control; because of the efficiency of the combination of these biostimulants in plant growth, it is to be expected that their use together adds more reproducible benefits for agricultural production than their separate use.

## Author Contributions

All authors listed have made a substantial, direct and intellectual contribution to the work, and approved it for publication.

## Conflict of Interest

The authors declare that the research was conducted in the absence of any commercial or financial relationships that could be construed as a potential conflict of interest.

## Publisher’s Note

All claims expressed in this article are solely those of the authors and do not necessarily represent those of their affiliated organizations, or those of the publisher, the editors and the reviewers. Any product that may be evaluated in this article, or claim that may be made by its manufacturer, is not guaranteed or endorsed by the publisher.
